# Pulmonary Paragonimiasis: A Case Series

**DOI:** 10.31729/jnma.8080

**Published:** 2023-03-31

**Authors:** Pratima Shah, Rinku Sah, Saugat Pradhan, Priyanka Bhandari, Ratna Baral, Basudha Khanal, Robin Maskey, Narayan Raj Bhattarai

**Affiliations:** 1 Department of Microbiology, B.P. Koirala Institute of Health Sciences, Dharan, Sunsari, Nepal; 2 Department of Internal Medicine, B.P. Koirala Institute of Health Sciences, Dharan, Sunsari, Nepal

**Keywords:** *case reports*, *eosinophilia*, *paragonimiasis*, *pleural effusion*

## Abstract

Paragonimiasis contributes to significant foodborne zoonosis worldwide. The major mode of transmission in humans is by consumption of uncooked or undercooked crabs and crayfish harbouring *Paragonimus metacercariae.* The infection begins with the symptoms like fever and lower respiratory involvement from a few months to a year, mimicking those of tuberculosis and leading to diagnostic delay. Here, we report two cases of paragonimiasis during a period of nine months. Both the cases presented with symptoms of productive cough with rusty sputum, chest pain, along with eosinophilia, and pleural effusion and had a history of consumption of smoked crab from the local river. The diagnosis was established by microscopic demonstration of *Paragonimus* ova in the sputum. Both patients were treated with praziquantel and recovered. Indeed, it is challenging to diagnose paragonimiasis due to the lack of its specific symptoms but should be considered in the differential diagnosis of eosinophilia and pleural effusion in such lung diseases.

## INTRODUCTION

Paragonimiasis commonly involving the lung and pleura is caused by several species of *Paragonimus* also called lung fluke. It is distributed worldwide and has been estimated that about 22.8 million people are at risk of infection globally. Most cases infected with *Paragonimus westermani* are consistently reported from Asia, Africa and South America because of cultural dietary customs of ingesting raw or undercooked crustacean harbouring metacercariae, the infective stage.^1^ However, few cases are reported from Nepal.^2^ Most of the cases are misdiagnosed due to similar clinical conditions which do not show specific symptoms. Therefore, cases of pulmonary paragonimiasis are discussed here.

## CASE REPORT

### CASE 1

A 39-year-old female, from the Bhojpur district of eastern Nepal, a previously healthy female presented with complaints of chest and neck pain for three months which was burning in character associated with dysphagia on and off, productive cough for fifteen days accompanied by blackish mucous flakes in sputum and dyspnea on walking up hills. In the beginning, she attempted a private hospital for medical advice where she reported marked eosinophilic leukocytosis and was prescribed steroids for two weeks which improved her symptoms. However, she was brought to the outpatient department of a tertiary care centre after two months as soon as she acquired symptoms again as mentioned above. Further repeated interrogation about her dietary habits revealed frequent consumption of smoked crabs collected from the river during the lockdown period due to COVID-19.

**Figure 1:**
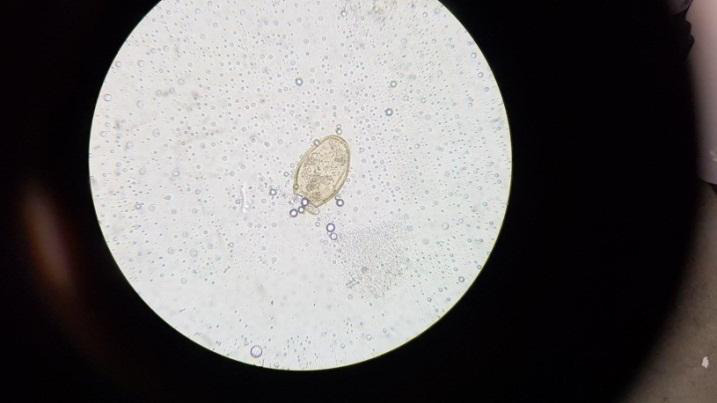
Wet mount preparation of sputum sample showing Paragonimus egg in case 1

On examination, she was afebrile, had a blood pressure of 130/90 mmHg, heart rate of 88 beats/minute, respiratory rate of 28/minute, and saturating well in ambient air (peripheral SpO2 98%). There was, however, decreased vocal fremitus, diminished breath sounds and a stony dull percussion note over the right and left infra-axillary and infrascapular region of the chest. There were no other added sounds heard. She was admitted for further evaluation and investigation. The chest X-ray revealed bilateral pleural effusion, and complete blood count (CBC) showed leukocytosis, 15,200 cells/mm3 with 40% eosinophils and raised erythrocyte sedimentation rate (ESR) with no other obvious abnormalities in blood investigations. Sputum for acid-fast bacilli (AFB), fungal stains and cultures done were negative. The sputum microscopy on wet mount preparation for parasitic examination showed characteristic operculated eggs of *P. westermani* (Figure 1). Stool routine microscopy did not show any significant findings.

### CASE 2

The second case admitted in the hospital was a 55 years male from Bhojpur, who presented with productive cough for 3-4 months with hemoptysis, throat irritation generalised itching all over the body which was on and off for the same duration. The patient gave a history of a similar kind of illness in the past around five months back and was admitted to other hospitals in view of hypereosinophilic syndrome and was managed with steroids which relieved his symptoms. He had a history of eating undercooked crab and frogs caught from the river.

**Figure 2:**
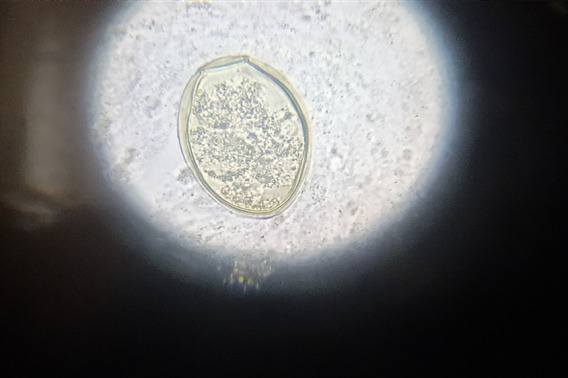
Wet mount preparation of sputum sample showing Paragonimus egg in case 2.

On the day of admission, clinical findings of this patient were as follows: temperature of 99F, heart rate of 80 beats/minute, blood pressure 160/90 mmHg, respiratory rate of 18 breaths/minute and multiple papular lesions over the body. His ultrasonography of the abdomen revealed mild ascites and mild bilateral pleural effusion. Contrast-enhanced computed tomography (CECT) chest, abdomen and pelvis revealed simple pulmonary eosinophilia and eosinophilic enteritis. Initial complete blood count revealed leukocytosis, 24600 cell/mm^3^, eosinophil-65%. Peripheral blood smear examination revealed eosinophilia (77%), absolute eosinophil count (AEC)-16810 cells/ul. Other blood investigations were within normal limits. The sputum test was negative for acid-fast bacilli (AFB), fungal and bacterial culture. Microscopic investigation (wet mount preparation) of sputum finally detected operculated eggs of *P. westermani*. Additional microscopic stool examinations for parasitic infestation were negative (Figure 2).

## DISCUSSION

Here, we report two cases of pulmonary paragonimiasis from eastern Nepal which documents the existence of disease in this region but often overlooked in the absence of proper diagnostic facilities. So far, only a few cases of paragonimiasis have been reported from other parts of Nepal because of limited diagnostic capacity and frequent misdiagnosis with the nonspecific symptoms. This extends to multiple hospital visits, admissions and unnecessary treatment with antibiotics and ATT. Paragonimiasis is an infection caused by lung flukes of the genus Paragonimus encompasses about 40 several species. Although Paragonimus species are widely distributed globally, human infections remain undetermined. Human infection is most often manifested as pulmonary and pleuropulmonary paragonimiasis of insidious onset, but abdominal, pericardial and cerebral paragonimiasis rarely occur. Most cases of paragonimiasis occur in China, Japan, Korea, Taiwan, Philippines, Nepal, Bangladesh, Myanmar, and Thailand, and are caused by Paragonimus westermani.^3,4^

Human paragonimiasis requires three hosts, snails, crustaceans and humans to complete its life cycle. The life cycle begins when eggs are shed from sexually competent adult worms that inhabit the lung tissue into bronchioles. The eggs are excreted via sputum or faeces and passed outside to the environment. In freshwater, eggs hatch to release free-swimming miracidia which invade the first intermediate host snails and eventually give rise to cercariae. These cercariae enter the second intermediate hosts, crab or crayfish in which they encyst as the infective stage, metacercariae. Ingestion of raw, undercooked, smoked or alcohol-pickled fresh water crabs or crayfish bearing metacercaria by humans completes the cycle of infection. The metacercariae exocyst in the duodenum penetrates the gut wall to migrate up through tissues to the pleural cavities and lung and form a capsule in which the adult pair resides and produce eggs.^5,6^

Generally, the estimated time taken from infection to laying eggs is 65 to 90 days with a life span of 1 to 20 years in humans. The signs and symptoms are seen in paragonimiasis result from the early migration of metacercariae from the small intestine to the lungs. Therefore, the patient may develop a migratory allergic skin rash.^4^ In the majority of the cases presenting symptoms commonly include productive cough with bloody sputum (77.9%), nonproductive cough (1.5%) and chest pain (67.6%).^6^ Both the patients presented with similar common symptoms of productive cough with hemoptysis and chest pain which mimics pneumonia and pulmonary tuberculosis that is endemic in our population and commonly misdiagnosed. Further investigations revealed eosinophilic leucocytosis but sputum for AFB was negative. Therefore, they were treated with steroids for hypereosinophilia. A few months later, they developed the symptoms again for which the patients were investigated up to a point at our centre.

Patients with human paragonimiasis exhibit a wide variety of nonspecific findings on physical examination, chest radiographs, CT scans, and blood investigations. However, definitive paragonimiasis diagnosis classically is based on viewing Paragonimus eggs or parasites in sputum, faeces, bronchoalveolar lavage (BAL), lung or pleural biopsy specimens or bodily fluids by microscope.^6^

Several studies have shown Paragonimus ova in 55.6 to 72% of the sputum specimens,^1,8^ and 11%-15% for a stool sample.^9^ A radiological finding of pleural effusion in patients with pleuropulmonary paragonimiasis has been reported to vary from 2.9% to 69%. Leucocytosis and eosinophilia in blood and pleural effusion is a supportive laboratory findings.^10^ In our patients, an accurate diagnosis of *P. westermani* infection was made on discovering oval, yellowish-coloured operculated eggs in their sputum though eggs were not detected in stool specimens accompanied by bilateral pleural effusion and peripheral blood leukocytosis and eosinophilia. For the treatment of this disease, WHO has recommended praziquantel and triclabendazole. The drug of choice is praziquantel at 25 mg/kg thrice daily for three days with high efficacy of 80-90%. All the manifestations of the patients promptly settled down in a few days after receiving treatment as per recommendation.^1,3^

The present cases are reported from eastern Nepal which documents the existence of the disease in this region but is often overlooked in the absence of proper diagnostic facilities. So far, only a few cases of paragonimiasis have been reported from other parts of Nepal because of limited diagnostic capacity and frequent misdiagnosis with non-specific symptoms. This extends to multiple hospital visits, admissions and unnecessary treatment with antibiotics and antitubercular therapy. This report also calls attention to enquire about the local customs of eating raw or undercooked crayfish or crab in all patients with undefined cough with or without hemoptysis, chest pain, eosinophilia and pleural effusions and considers the possibility of paragonimiasis. Detection of such cases further demands the introduction of diagnostic facilities where the customary eating habits of the intermediate habitat of the parasites prevail in the community.

## Consent:

**JNMA Case Report Consent Form** was signed by the patient and the original is attached with the patient chart.
